# Editorial: Changes in food functional components during innovative processing technologies and delivery systems, digestion, and metabolism

**DOI:** 10.3389/fnut.2023.1200010

**Published:** 2023-06-01

**Authors:** Lianliang Liu, Jin Wang, Zhongbin Deng

**Affiliations:** ^1^State Key Laboratory for Managing Biotic and Chemical Threats to the Quality and Safety of Agro-products, Key Laboratory of Animal Protein Deep Processing Technology of Zhejiang, Zhejiang-Malaysia Joint Research Laboratory for Agricultural Product Processing and Nutrition, School of Food and Pharmaceutical Sciences, Ningbo University, Ningbo, Zhejiang, China; ^2^Key Laboratory of Environmental Medicine and Engineering, Ministry of Education, Department of Nutrition and Food Hygiene, School of Public Health, Southeast University, Nanjing, China; ^3^Division of Immunotherapy, Department of Surgery, Brown Cancer Center, University of Louisville, Louisville, KY, United States

**Keywords:** innovative processing technologies, delivery systems, digestion, metabolism, functional food

Active peptides, phenolic acid, polysaccharides, oligosaccharides, exopolysaccharides, free amino acids, organic acids, and vitamins are classified as functional food components, which actively function to decrease and eliminate microbes, oxidation, and hypersensitivities of ingested foods.

Innovative technologies recently developed have shown abilities to reduce the damage to functional food components, compared to traditional food processing methods. These new technologies include pulsed electric field, microwave, ohmic heating, ultrasound, high pressure, and subcritical water/supercritical fluid methods (1, 2).

Besides, it has focused on embedding the functional components in macromolecules to enhance the bioavailability of functional food components. Proteins, polysaccharides, dietary fiber, pectin, and polyunsaturated fatty acids are used as macromolecules to protect the functional components from degradation that occurs during digestion and metabolism (3).

In this collection, a total of four papers were published. Zhao et al. found that dihydromyricetin and myricitrin, as main active compounds in the vine tea, synergistically inhibited the tyrosinase activity in B16F10 cells and could regulate signaling pathways such as melanogenesis, NF-κB, and apoptosis. Accordingly, it was beneficial to applying low-toxicity vine tea extract for the prevention and treatment of melanoma (Zhao et al.). Packaging material can influence the quality of food products during transportation. The microbial variation on bigeye tuna (*Thunnus obesus*) has been evaluate based on the effects of the packaging material to maintain a high quality product during transportation. Cheng et al. proved aluminum foil paper packaging could efficiently prevent TVB-N, redness value, metmyoglobin content increase to keep better color, and flavor of bigeye tuna. Moreover, aluminum foil had excellent barrier properties on water, oxygen, light, and microorganisms, which were the main factors causing food spoilage. Thereby, aluminum foil paper packaging could effectively inhibit microbial diversity and growth (such as *Pseudomonas*), guaranteeing the quality of bigeye tuna preferably during transportation (Cheng et al.).

Traditional hot processing methods, such as boiling treatment, affected protein digestibility with the degree of doneness in the meat. Yu et al. showed that heat processing reduced the protein digestibility and the bioavailability of amino acids, which had a negative influence on the nutritional values of meat. Besides, it was found that the digestibility of meat protein in the inner layer was higher than middle and outer layers (Yu et al.). On the other hand, non-thermal processing techniques, such as cold plasma processing, have garnered a great deal of attention in the food processing industry. However, debate remains as to whether protein oxidation and heme degradation occurs with cold plasma processing. Wang et al. indicated that dielectric barrier discharge cold plasma promoted protein oxidation, changed the secondary structure of protein, and accelerated lipid oxidation.

Stimulus-responsive smart hydrogels prepared by polysaccharides are used as targeted delivery systems for bioactive substances, such as curcumin, quercetin, and probiotics, helping to control the release rate and improve intestinal stability during their digestion and metabolism (4). These cases show that innovative processing technology and delivery system can improve the functional properties of functional food components by maintaining the integrity of their structure, compared with traditional processing technologies according to the changes of functional food components in processing, transportation, digestion, and metabolism.

New processing techniques and delivery systems reduce the disruption of the original compositional structure and functional properties of foods. In the meanwhile, the quality and nutritional integrity should to be achieved. However, related work in the future should focus more on the following items:

(i) The effect of changing the operation time and intensity of new processing technology on the nutrition and shelf life of food functional ingredients.
(ii) The effect of using non-heating techniques on the sensory properties of food while ensuring time and energy. Further exploration is needed to achieve a balance between quality and sensory properties.
(iii) Interactions between functional components of foods using innovative processing techniques or delivery systems and their impact on bioavailability.
(iv) In food delivery system, compare the advantages of gel and emulsion in delivering functional ingredients of food, and explore the future development direction of targeted delivery.

## Author contributions

All authors listed have made a substantial, direct, and intellectual contribution to the work and approved it for publication.
